# Are cluster-based psychological profiles of body investment, self-perception and loneliness associated with eating-disorder severity, global psychopathology, and self-injury?

**DOI:** 10.3389/fpsyg.2026.1748410

**Published:** 2026-05-13

**Authors:** Marco Scotto Rosato, Marzieh Abdoli, Margherita Stabile, Fabio Carraturo, Walter Milano, Stefania Cella

**Affiliations:** 1 Observatory on Eating Disorders, Department of Psychology, University of Campania “Luigi Vanvitelli”, Caserta, Italy; 2 UOSD Eating Disorder Unit, Mental Health Department ASL Napoli 2 Nord, Napoli, Italy

**Keywords:** body investment, cluster analysis, eating disorders, loneliness, psychopathology, self-esteem, self-harming behaviors

## Abstract

**Introduction:**

Patients suffering from eating disorders (EDs) often report low self-esteem, body shame, and experiences of social or emotional loneliness. The present study aimed to identify distinct psychological profiles in female adolescents and young adults with EDs and to examine their associations with ED severity, global psychopathology, and self-injurious behaviors.

**Methods:**

Thirty-eight female patients (age range 14–35; M_age_  = 20.47, SD_age_ = 5.59) attending an outpatient eating disorder service in southern Italy completed a sociodemographic questionnaire and self-report measures assessing self-esteem, body shame, social and emotional loneliness, affective body investment, psychopathology, and eating disorder severity. A semi-structured interview was administered to assess self-injurious behaviors.

**Results:**

Cluster analysis identified three profiles: (1) Vulnerable–Over-Invested, characterized by low self-esteem, high body shame, and high social and emotional loneliness, along with low body image and elevated body care and touch; (2) Vulnerable–Compensated, with higher self-esteem, lower body shame and loneliness, and high overall body investment; and (3) Vulnerable–Detached, characterized by low body touch, care, and protection, and high social and emotional loneliness. Significant differences among profiles emerged in ED severity (*F*(2, 35) = 4.24, *p* = .01, *R^2^* = .27) and self-injurious behaviors (*χ^2^*(2) = 8.20, *p* = .017), with the Vulnerable–Over-Invested group showing greater clinical impairment. No significant differences emerged in global psychopathology (*F*(2, 35) = 1.02, *p* = .39, *R^2^* = .08).

**Discussion:**

Overall, the findings suggest heterogeneous psychological profiles in this clinical sample of women with eating disorders. Given the cross-sectional design and small sample size, the results should be interpreted cautiously as descriptive associations requiring replication.

## Introduction

Eating disorders (EDs) are serious psychiatric conditions that can significantly compromise physical and mental health, and overall quality of life (De La Rie et al., 2005). Symptomatic manifestations are not limited to eating habits and behaviors, but also extend to beliefs about oneself, affective regulation, and interpersonal functioning (Arcelus et al., 2013). Furthermore, EDs are often associated with impaired self-evaluation and the way in which individuals perceive and relate to their bodies (Abdoli et al., 2025). Self-esteem is recognized as one of the fundamental risk and maintenance factors for eating disorders. Self-esteem is an overall evaluation of an individual’s self-worth. When compromised, low self-esteem contributes to the formation of a fragile and unstable sense of adequacy, an individual vulnerability that makes individuals more sensitive to criticism and external evaluations, and more unsatisfied with their own body and appearance (Hatoum et al., 2024; Mora et al., 2022).

Empirical studies suggest that lower self-esteem is associated with higher body dissatisfaction and disordered eating behaviors, including dietary restraint and compensatory behaviors (Mina et al., 2021).

Body shame, on the other hand, is defined as the affective manifestation of perceived inadequacy embodied in the self. According to empirical research, body shame often mediates between compromised self-esteem and the severity of eating symptoms (Iannaccone et al., 2016). Individuals who experience a strong sense of body shame tend to internalize ideals of appearance and control their bodies as a means of restoring coherence and self-acceptance (Bi et al., 2024).

In this perspective, the body becomes the field in which adequacy and personal value are negotiated. Attempts to regulate, for example through body care or social withdrawal, perform both defensive and compensatory functions (Cella and Cotrufo, 2023).

To this end, the concept of body investment describes how individuals relate to their bodies, in terms of care, protection, physical contact, and attitudes (Orbach and Mikulincer, 1998).

In clinical populations that report individual vulnerability and intense feeling of shame, manifestations of high levels of body care and monitoring may represent defensive control rather than a genuine investment in the body (Fuchs, 2022). Conversely, poor care and avoidance of physical contact may indicate withdrawal and disembodiment, which serve as protective mechanisms against shame (Piran, 2016).

Interpersonal factors are also relevant. Feelings of isolation and social disconnection relate to greater symptom severity, and they can follow shame and withdrawal (Arcelus et al., 2013; Meneguzzo et al., 2024). These factors are associated with EDs psychopathology and body shame (Mason, 2024). Withdrawing from relationships can be an attempt to avoid exposure and judgment, which reinforces cycles of shame and focus on appearance.

Although these aspects have been investigated separately, only a few empirical studies have integrated self-esteem, body shame, body investment, and loneliness into a single theoretical framework. These variables connect self-beliefs, body-related emotion and practice, and social connection with processes described in existing models of eating-disorder maintenance. Moreover, most studies have adopted var*iable-centered* approaches, focusing on average associations rather than person-level configurations.

Variable-centered analyses test average relations among variables, but they can mask subgroups that combine traits in distinct ways. Person-centered methods are designed to identify these subgroups using the joint pattern of indicators in each person (Howard and Hoffman, 2018).

Cluster analysis is particularly effective in identifying clinical subtypes as it allows patients to be grouped based on multiple characteristics, such as clinical symptoms or psychological characteristics. This methodology highlights the typical heterogeneity of eating disorder patients. For example, according to recent empirical research involving this population (Camacho-Barcia et al., 2025; Li et al., 2023; Meneguzzo et al., 2025), differences in terms of symptoms, therapeutic outcomes, and emotional skills have been deepened.

The present study aimed to identify distinct psychological profiles in a clinical sample of female adolescents and young adults with eating disorders, based on self-esteem, body shame, body investment, and loneliness.

We hypothesized that (1) clustering these variables would yield distinct profiles reflecting different ways of regulating inadequacy and shame; (2) profiles marked by low self-esteem, high body shame, and high loneliness would show higher ED severity and self-harming behaviors; and (3) profiles marked by compensatory or detached body investment would differ in severity, reflecting different defensive organizations of vulnerability.

## Methods

### Participants and procedures

For the present study, 38 female patients (Age range 14–35, M = 20.47, SD = 5.59) attending a territorial ambulatory service for the treatment of eating disorders in southern Italy were involved. Participants were assessed during the initial phase of clinical care, following the first psychiatric evaluation. The psychiatric evaluation and clinical diagnosis were established by the psychiatrist in charge of the service using the DSM-5 criteria (American Psychiatric Association, 2013). The following inclusion criteria were used: (a) diagnosis of eating disorders; and (b) absence of neurological (including neurodevelopmental and neurocognitive), psychotic, or schizophrenia spectrum disorders. In addition, interested participants and their parents/legal guardians—in the case of minors—following a detailed explanation of the procedures and purposes of the project, were required to sign an informed consent form. The study was conducted in accordance with the ethical principles of the Declaration of Helsinki (World Medical Association, 2013) and all procedures performed were approved by the Ethics Committee of the University of Campania “Luigi Vanvitelli.”

Participants completed a sociodemographic questionnaire and self-report measures assessing self-esteem, body shame, loneliness, affective investment in the body, eating disorder severity, global psychopathology and a semi-structured interview to explore self-injurious behaviors.

### Measures

The sociodemographic sheet is an *ad hoc* questionnaire assessing age, gender, occupational and marital status, family background, sexual orientation and lifestyle, body modification, and physical activity. Body modification items (tattoos, piercings, cosmetic surgery, and intentions) were included as exploratory indicators of body-related practices and because prior studies have examined body modifications in relation to eating disorder symptoms and self-injurious behaviors (Iannaccone et al., 2013; Preti et al., 2006).

Self-esteem was assessed using the Rosenberg Self-esteem Scale (Rosenberg, 2015). It consists of ten items on a 4-point Likert scale, ranging from “Strongly agree” to “Strongly disagree.” Higher scores indicate greater self-esteem. The scale has been validated in the Italian sample (Prezza et al., 1997) and has demonstrated consistent reliability and validity (Rosenberg, 2015); in our sample, the internal consistency coefficient was 0.83.

Body shame was assessed using the Bodily Shame subscale of the Experience of Shame Scale (ESS; Andrews et al., 2002), validated in Italian samples (Velotti et al., 2018). It is composed of four items on a 4-point Likert scale from “Not at all” to “Very much,” with higher scores indicating higher body shame. The measure demonstrated acceptable psychometric properties (Andrews et al., 2002); in the present sample, Cronbach’s alpha = 0.74.

The De Jong Gierveld Loneliness Scale (De Jong-Gierveld and Kamphuis, 1985) was used to measure emotional and social loneliness. The instrument consists of eleven items rated on a 5-point Likert scale, ranging from “None of the time” to “All of the time”. A higher score indicates a greater sense of loneliness. The Italian version has shown adequate psychometric properties in previous studies (Zammuner, 2008); in the present study, the reliability coefficients were 0.70 for both emotional and social loneliness scales.

The Body Investment Scale (BIS; Orbach and Mikulincer, 1998) was used to assess emotional investment in own body. The instrument consists of twenty-four items rated on a five-point Likert scale. It consists of four subscales: feelings and attitudes toward the body (Body Image); Body Care; Body Protection; and comfort with physical contact (Body Touch). Higher scores indicate higher emotional investment in the body. The BIS has demonstrated solid psychometric properties (Cella and Cotrufo, 2023); in the present study, the reliability coefficients were 0.87 for Body Image, 0.61 for Body Care, 0.49 for Body Protection and 0.71 for Body Touch scale. An Italian translation of the instrument has been used in previous studies conducted in Italian samples (Abdoli et al., 2025; Cella et al., 2021; Cipriano et al., 2020).

The Eating Disorder Risk Composite (EDRC) scale from the Eating Disorders Inventory-3 (EDI-3; Garner, 2004) was administered. It is the composite score of “Drive for thinness,” “Bulimia” and “Body Dissatisfaction” scales, consisting of 25 items on a five-point Likert scale ranging from “Always” to “Never.” According to the author, the tool can be used to assess the severity of the eating disorder symptomatology. It has an Italian adaptation and validation and has demonstrated solid psychometric properties (Rosenberg, 2015; Garner, 2004); in our sample, Cronbach’s alpha was 0.78.

The Symptom Checklist-90 (SCL-90; Derogatis et al., 1973) is a self-report instrument used to assess a wide range of psychological and psychiatric symptoms. It consists of 90 items that explore various psychological symptoms. Individuals rate the degree to which they have experienced each symptom during a given period of time on a 5-point Likert scale, ranging from 0 (“Not at all”) to 4 (“Extremely”). Scores of 1 or higher indicate clinically relevant symptoms. The SCL-90 has an Italian adaptation and validation and has demonstrated construct and convergent validity. Only the overall psychopathology scale was used in the present study, with Cronbach’s alpha = 0.98.

The Deliberate Self-Harm Inventory (DSHI; Gratz, 2001), Italian adaptation (Monti and D’Agostino, 2010) was administered during a semi-structured interview. The instrument assesses 16 different types of self-injurious behaviors. For each item, patients were asked if they had ever engaged in the specific behavior, as well as its frequency, duration, and severity.

### Statistical analysis

Cluster analysis was performed to identify psychological profiles by investigating the psychological aspects of body image, affective physical touch, body care, body protection, self-esteem, body shame, and emotional and social loneliness. Hierarchical cluster analysis was first performed using Ward’s method with Euclidean squared distance to explore the optimal number of clusters. The dendrogram was visually inspected to inform the selection of the cluster solution (see supplementary material). The final solution was refined using K-means clustering. Cluster validity was assessed using the Calinski–Harabasz index and cluster stability was evaluated using bootstrap resampling with Jaccard similarity coefficients. One-way ANOVA was used to confirm that the identified profiles differed significantly on the psychological dimensions used to derive them. A sensitivity analysis was conducted by repeating the clustering procedure without the Body Protection scale due to its low internal consistency. To assess which psychological profile was most associated with more severe eating psychopathology and more severe global psychopathology, multiple regression analyses were conducted, controlling for age. Regression assumptions were assessed through inspection of residual plots and variance inflation factors to evaluate multicollinearity. Bootstrapping (5,000 samples) was used to obtain robust confidence intervals. Finally, a chi-square test was used to examine the relationship between psychological profile and involvement in self-harming behaviors. Expected cell counts were examined, and likelihood ratio statistics were also reported given the small sample size. Statistical analyses were conducted using IBM SPSS Statistics, version 29 for macOS. Goodness-of-fit indices were run in R (version 4.5.0) via RStudio (version 2025.05.0 + 496) using *cluster, clusterSim, factoextra, fpc,* and *ggplot2* packages. Variables were standardized within the clustering procedure to ensure comparability across scales. Statistical significance was set at *p* < 0.05. A sensitivity power analysis conducted using G*Power indicated that the study was able to detect medium-to-large effect sizes. Statistical power was set at 0.80 (Cohen, 2013), *N* = 38, *α* = 0.05.

## Results

### Characteristics of the sample

The sample consisted of 38 female patients attending an outpatient clinic for ED treatment in southern Italy. Participants’ ages ranged from 14 to 35 years (*M* = 20.47, SD = 5.59). Most of the sample (*N* = 25, 69.4%) were students; 11.1% (*N* = 4) were employees, 5.6% (*N* = 2) were self-employed, and 13.9% (*N* = 5) were unemployed or not in employment. Only one participant was married (*N* = 1, 2.9%), while the remaining 33 (97.1%) were single.

Regarding sexual orientation, 83.8% (*N* = 31) identified as heterosexual, 13.5% (*N* = 5) as bisexual, and one participant (2.7%) as homosexual. Half of the sample (*N* = 19, 50%) reported having had sexual intercourse, and 26.3% (*N* = 10) were currently in a stable relationship. Ten participants (26.3%) reported choosing not to have sexual intercourse, and most (*N* = 37, 97.4%) reported not changing partners frequently.

Fourteen participants (36.8%) reported attending a gym regularly, for an average of 4.43 h per week (SD = 2.32, range = 1–10). The main motivations were keeping fit (*N* = 8, 57.1%), losing weight (*N* = 4, 10.5%), and increasing muscle mass (*N* = 2, 5.3%). Most participants (*N* = 33, 86.8%) reported being careful not to gain weight and expressed a desire to lose approximately 6 kg (SD = 3.63).

Regarding body modifications, 23.7% (*N* = 9) had piercings and 34.2% (*N* = 13) intended to get one. Eighteen-point 7 % (*N* = 7) already had at least one tattoo, while 60.5% (*N* = 23) expressed the intention to get one in the future. Two participants (5.3%) had undergone cosmetic plastic surgery, and 18.4% (*N* = 7) reported an intention to do so.

Concerning family background, 13.5% (*N* = 5) of mothers held an elementary school diploma, 37.8% (*N* = 14) a middle school diploma, 35.1% (*N* = 13) a high school diploma, and 13% (*N* = 5) a university degree. Regarding fathers, 5.6% (*N* = 2) held an elementary school diploma, 44.4% (*N* = 16) a middle school diploma, 33.3% (*N* = 12) a high school diploma, and 11% (*N* = 4) a university degree. Most participants (*N* = 29, 76.3%) reported that their parents were married, 15.8% (*N* = 6) divorced, 5.3% (*N* = 2) separated, and 2.6% (*N* = 1) widowed. See Table 1.

**Table 1:** Characteristics of the sample.

Variable	Category	*N*	%
Occupational status	Students	25	69.4%
	Employees	4	11.1%
	Self-employed	2	5.6%
	Unemployed/not in employment	5	13.9%
Marital status	Single	33	97.1%
	Married	1	2.9%
Sexual orientation	Heterosexual	31	83.8%
	Bisexual	5	13.5%
	Homosexual	1	2.7%
Sexual activity	Had sexual intercourse	19	50.0%
	In stable romantic relationship	10	26.3%
	Abstinent by choice	10	26.3%
	Change partners frequently	1	2.6%
Gym attendance	Regularly attend gym	14	36.8%
	Goal: keep fit	8	57.1% (on 14)
	Goal: lose weight	4	28.6% (on 14)
	Goal: increase muscle mass	2	14.3% (on 14)
Weight concern	Careful not to gain weight	33	86.8%
Body modifications	Piercings	9	23.7%
	Intend to get piercing	13	34.2%
	Tattoos	7	18.7%
	Intend to get tattoo	23	60.5%
	Had cosmetic surgery	2	5.3%
	Intend cosmetic surgery	7	18.4%
Mother’s education	Elementary school	5	13.5%
	Middle school	14	37.8%
	High school	13	35.1%
	University degree	5	13.0%
Father’s education	Elementary school	2	5.6%
	Middle school	16	44.4%
	High school	12	33.3%
	University degree	4	11.0%
Parents’ marital status	Married	29	76.3%
	Divorced	6	15.8%
	Separated	2	5.3%
	Widowed	1	2.6%

### Cluster analysis

**Figure 1:**
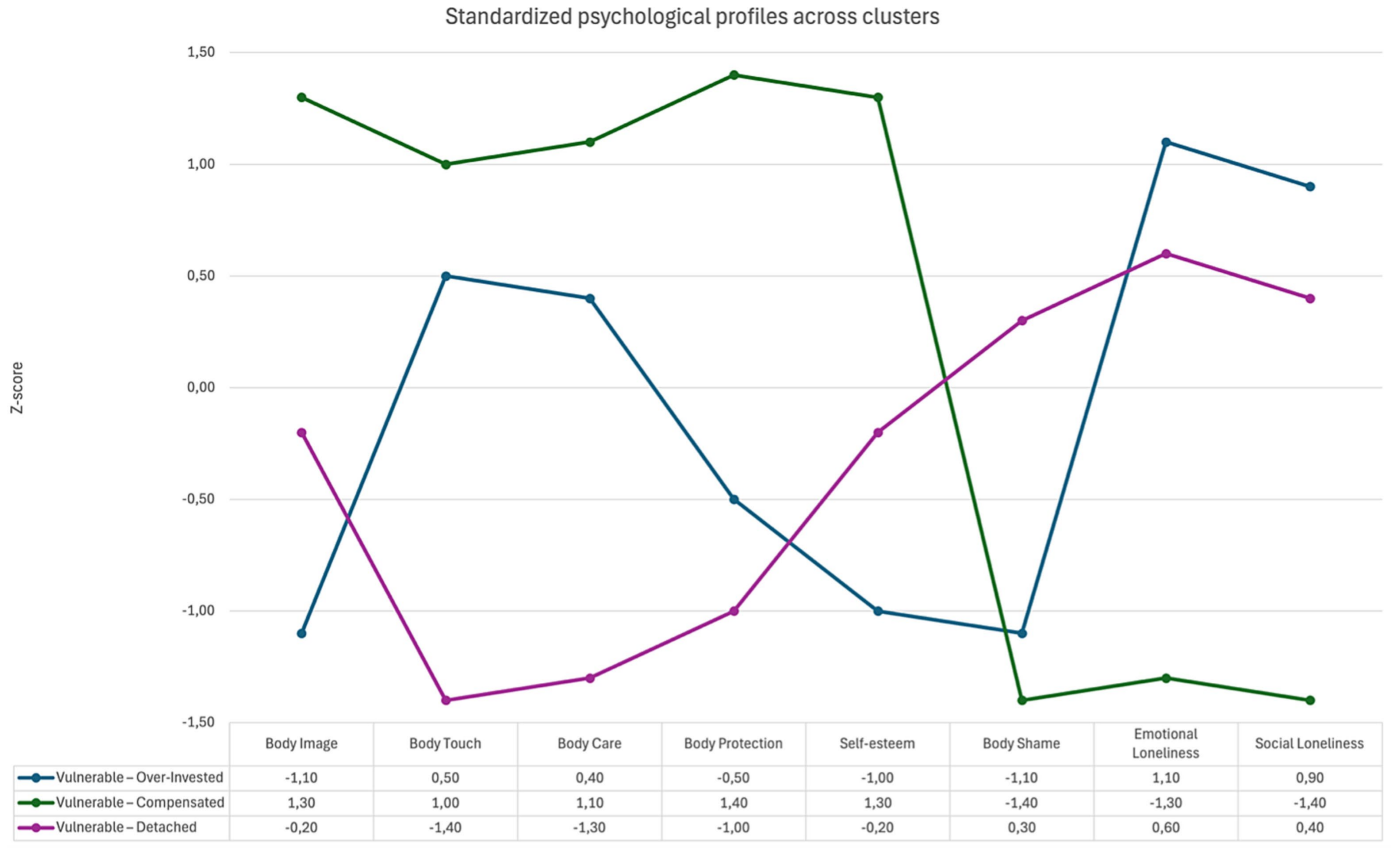
Standardized psychological profiles across clusters. Higher scores indicate greater body investment, self-esteem, body shame, and loneliness.

Dendrogram and hierarchical cluster analysis (Ward’s method, squared Euclidean distance), conducted on standardized scores of self-esteem, body shame, emotional and social loneliness, and the four dimensions of the Body Investment Scale, revealed three distinct psychological profiles (Figure 1). The Calinski-Harabasz index reached its maximum value for the three-cluster solution. Jaccard coefficients showed values of 0.72, 0.74, and 0.63 for the three clusters (5,000 bootstraps), indicating moderate to good stability of membership. Mean scores and ANOVA results are reported in Table 2.

**Table 2:** Mean scores and ANOVA results of the clustering variables across groups.

Variable	Vulnerable over-invested (*N* = 15)		Vulnerable compensated (*N* = 8)		Vulnerable detached (*N* = 15)		*F* (2,35)	*p*
	M	SD	M	SD	M	SD		
Body image	1.63	0.52	3.39	0.76	2.34	1.09	11.68	< 0.001
Body touch	3.38	0.66	3.56	0.39	2.52	0.7	9.70	< 0.001
Body care	3.71	0.5	4.02	0.46	2.94	0.64	12.17	< 0.001
Body protection	3.53	0.85	4.1	0.2	3.4	0.43	3.67	0.036
Self-esteem	6.20	3.03	18.25	4.92	10.93	4.64	22.22	< 0.001
Body shame	15.0	1.13	12.0	3.74	13.87	2.0	4.79	0.014
Emotional loneliness	4.8	1.15	2.25	1.91	4.67	1.34	9.88	< 0.001
Social loneliness	3.07	1.49	1.5	1.6	3.47	1.46	4.65	0.016

The profile “Vulnerable—Over-Invested” (*N* = 15; 39.5%) combines low Body Image and low self-esteem with high body shame and high emotional and social loneliness. At the same time, Body Care and Body Touch are relatively higher than in the other clusters. In practical terms, this profile pairs negative self-evaluation and social–emotional distress with increased body-focused behaviors.

The profile “Vulnerable—Compensated” (*N* = 8; 21%) shows higher self-esteem and higher Body Image, with lower body shame and lower emotional and social loneliness. BIS subscales are relatively higher overall, suggesting more positive attitudes toward the body and more care and comfort with contact. This is the least impaired pattern among the three, within this clinical sample.

The profile “Vulnerable—Detached” (*N* = 15; 39.5%) shows moderate Body Image, self-esteem, and body shame, but relatively higher loneliness. Body Touch, Body Care, and Body Protection are lower than in the other clusters. In practical terms, this profile pairs social–emotional disconnection with reduced body-directed care and comfort with contact.

### Eating disorder psychopathology

To assess differences in the severity of the eating disorder among the different profiles, multiple regression analyses were conducted. Cluster membership was dummy coded, and age was used as covariate. Bootstrap with 5,000 samples was used to obtain robust estimates. The regression model was significant, indicating that the psychological profiles differed in ED psychopathology (F (Abdoli et al., 2025; Gratz, 2001) = 4.24, *p* = 0.012, *R^2^* = 0.27). In particular, the profile “Vulnerable—Over-Invested” showed significantly higher levels of eating disorder severity than the profile “Vulnerable—Compensated” [*β* = −26.16; 95% CI (−50.74, −4.99)] and the profile “Vulnerable—Detached” [β = −15.46; 95% CI (−29.91, −4.73)] while the differences between “Vulnerable—Detached” and “Vulnerable—Compensated” were not significant [β = −10.7; 95% CI (−36.81, 20.45)]. Age did not contribute significantly to the model (*p* = 0.95). Descriptive statistics are presented in Table 3.

**Table 3:** EDRC scores and 95% confidence intervals by cluster.

Cluster	Eating disorder risk composite score		95% CI
	M	SD	
Vulnerable—over-invested (*N* = 15)	69.48	4.61	[60.1, 78.9]
Vulnerable—compensated (*N* = 8)	43.32	6.88	[29.3, 57.3]
Vulnerable—detached (*N* = 15)	54.02	4.7	[44.5, 63.6]

### Global psychopathology

The multiple regression analyses performed to evaluate differences in the global severity index of psychopathology across profiles, highlighted no significant differences [F (Abdoli et al., 2025; Gratz, 2001) = 1.02, *p* = 0.39, *R^2^* = 0.08]. Cluster membership was dummy-coded, age was entered as covariate and all models were bootstrapped (5,000 samples, 95% CI) to ensure robust estimates. Mean score, standard deviation and confidence intervals are presented in Table 4.

**Table 4:** Global severity index (SCL-90) scores and 95% confidence interval by cluster.

Cluster	Global severity index		95% CI
	M	SD	
Vulnerable—over-invested (*N* = 15)	2.04	0.67	[1.7, 2.4]
Vulnerable—compensated (*N* = 8)	1.06	0.63	[0.6, 1.5]
Vulnerable—detached (*N* = 15)	1.86	0.99	[1.3, 2.3]

### Self-harming behaviors

Findings from the DSHI administration revealed that 14 patients engaged in self-harming behavior. Specifically, ten of them reported intentionally cutting their wrists, arms, or other parts of their bodies without the intention of killing themselves. Eight patients used blows to hurt themselves, such as punching themselves or, in four cases, voluntarily banging their heads against something until they bruised or contused themselves. Seven patients reported scratching themselves to the point of bleeding or causing visible scars, while six of them used biting to the point of cutting their skin. Some patients (*N* = 4) reported sticking sharp objects into their skin, intentionally burning themselves (*N* = 3), or incising words into their skin (*N* = 2). Seven patients reported interfering with the wound healing process. One patient reported rubbing sandpaper on their body, and one patient reported trying to ingest bleach or toxic products. When asked, “What is your relationship with your scars?”, of the seven patients who had at least one, five reported feelings of shame.

Regarding the presence of self-harming behaviors across groups, the chi-square test showed a significant association between cluster membership and self-harming behaviors [*χ^2^*(2) = 8.20, *p* = 0.017; Likelihood ratio *χ^2^*(2) = 10.73, *p* = 0.005; Cramer’s V = 0.47], with the profile “Vulnerable—Over-Invested” being at greater risk (*N* = 9, 60%), compared to “Vulnerable—Detached” (*N* = 5, 33%) and “Vulnerable—Compensated” (*N* = 0, 0%). Results are summarized in Table 5.

**Table 5:** Self-harming behavior prevalence across groups.

Cluster	Yes	No	Prevalence
Vulnerable—over-invested (*N* = 15)	9	6	60%
Vulnerable—compensated (*N* = 8)	0	8	0%
Vulnerable—detached (*N* = 15)	5	10	33%
Total (*N* = 38)	14	24	36.8%

## Discussion

Three different psychological profiles were identified through a clinical sample of female adolescents and young adults with eating disorders. Although these profiles share a common ground of psychological vulnerability, this vulnerability appears to be organized primarily around a pervasive sense of inadequacy and body-related shame, which the three different psychological profiles manifest through different defensive configurations: overcontrol and hypervigilance toward the body, compensatory integration, or withdrawal and bodily detachment, a well-known pattern in literature, with empirical studies emphasizing the role of self-esteem and body shame in the ED psychopathology (Cella et al., 2020; Fairburn et al., 2003).

This study integrated various dimensions of individual vulnerability within a person-centered perspective, showing how different configurations can discriminate types of patients with ED in terms of defensive organizations and clinical severity, either regarding eating symptoms, overall psychological impairment, or use of self-harming behaviors.

The profile “Vulnerable—Over-Invested” is characterized by low levels of self-esteem, intense body shame, high levels of social and emotional loneliness, and a high level of body care and contact. It is a combination of variables that may underlie a general psychological vulnerability in which the body becomes the main arena where self-worth is negotiated. Although these patients devote attention to their body through monitoring and care, such behaviors appear to function less as genuine self-investment and more as defensive attempts to counter feelings of shame and internal fragmentation (Anzieu, 2017). These findings are also reported in empirical studies that associate low self-esteem and intense body shame to increased concern about physical appearance and to attempts to restore a sense of adequacy through body care or control (Abdoli et al., 2025; Woodward et al., 2019). Therefore, the body becomes the privileged means through which patients seek to regain control and coherence when experiencing intense feelings of shame and internal fragmentation. In this sense, the body is subjected to a process of discipline aimed at managing psychic tension rather than being imbued with vitality or eros, and emphasizing control, restriction, or meticulous care provides temporary relief from these feelings of inadequacy, while reinforcing the disconnection between bodily experience and affective life. This pattern characterizes individuals who exhibit the most severe symptoms of eating disorders and the highest prevalence of self-harming behaviors: in both cases, the body becomes the place where unmentalized affects are discharged and contained. It is a way to transform emotional pain and inadequacy into something controllable and tangible.

The profile “Vulnerable—Compensated” displays a relative adjustment compared to the other profiles, which cannot be interpreted in absolute terms. Participants in this profile report better levels of self-esteem and relationship with their bodies, both in terms of affection and shame. Levels of loneliness are lower. Even though these individuals are less compromised than those in the other profiles, they still represent a segment of the population with latent vulnerability. This relative adaptation can be read as a form of compensatory self-organization, in which the individual’s resources and adaptive defenses enable functioning despite underlying vulnerability (Fonagy and Target, 2000). These patients display a comparatively stable sense of self-efficacy and adequacy, which moderates the impact of vulnerability and allows better regulation of body-related experiences (Turk et al., 2021). This is clearly reflected in lower clinical impairment: this profile is associated with lower severity of eating-disorder symptoms and the absence of self-harming behaviors. When considering the global severity index of the general psychopathology, even if patients in this group reported lower scores, no significant differences emerged across groups, confirming that they are part of a clinically vulnerable population.

The profile “Vulnerable—Detached” is marked by moderate levels of self-esteem and body shame, high loneliness and poorer bodily investment, in terms of less care, contact, and protection of the body. This profile seems to be defined by a pervasive sense of inadequacy that is managed through withdrawal from bodily experience rather than through control, as in the “Vulnerable—Over-Invested” profile. Detachment from the body, both in empirical and theoretical terms, is recognized as a protective mechanism that allows the avoidance of shame experiences and exposure of vulnerability in relational contexts (McDougall, 1989; Mirabella et al., 2024; Tempia Valenta et al. 2025). It is a way of regulating emotional distress by minimizing bodily awareness and affective engagement, a defensive disembodiment: the body thus becomes silent, a background object rather than a lived experience. Despite this distancing, these patients maintain a residual sense of adequacy and relatively lower symptom severity, which can serve as partial protection against more serious psychopathological outcomes and the implementation of self-harming behaviors.

The use of cluster analysis allowed the identification of distinct clinically recognizable psychological organizations. Using this approach, it has been identified how feelings of inadequacy, shame, loneliness, and disconnection from the body can coexist in different ways in the various subgroups, offering a more nuanced understanding of vulnerability in the intake of patients with EDs.

The potential to differentiate these profiles demonstrates that vulnerability in these patients is not monolithic: patients may regulate inadequacy through hyper control, detachment, or finding a relative adaptation. Recognizing these elements provides clinically valuable information to calibrate therapeutic interventions to the individuality of the person.

This study is in line with existing research that has reported differentiated clusters in a sample of patients with eating disorders (e.g., 15, 34), but it stands out for the transversal domain of the variables considered. To date and to our knowledge, no study on this clinical sample has explored the domains of individual vulnerability, experiences of loneliness, and emotional investment in the body.

In conclusion, data show how fragility and relationship with the body can take different forms in eating disorders and how these “psychological profiles” are associated with different forms of clinical risk. These results highlight the centrality of feelings of inadequacy and shame expressed through the body: in the first profile through defensive bodily control, in the third through splitting and withdrawal from bodily experience, and in the second profile via relative adequacy and compensation.

The possibility of distinguishing these profiles offers relevant insights for therapeutic intervention. For subjects in the first profile, clinical work could focus on containing shame and building a more cohesive self, reducing the use of the body as a tool for regulation. In the third profile, on the other hand, intervention could aim to encourage a reconnection with emotions and bodily experiences, working on the recognition and mentalization of experiences. For the second profile, the goal is to consolidate existing resources in order to prevent regression in stressful situations. Across all profiles, interventions should emphasize the restoration of a sense of adequacy and personal value, rather than solely focusing on symptom reduction or body-related strategies.

Finally, although the cross-sectional design does not allow for causal inferences, these results underscore the importance of personalized therapeutic approaches that consider subjective specificities and the quality of bodily investment in identity construction.

## Conclusion

This study suggests that distinct patterns of body investment, self-esteem, and body-related emotions are associated with different levels of risk for eating disorders and self-injurious behaviors. Women with low self-esteem, high emotional and social loneliness, and elevated body shame appear most vulnerable. In contrast, those with high self-esteem and a nurturing relationship with their bodies are comparatively resilient. A third profile seems to cope through detachment from bodily sensations and heightened social sensitivity, reflecting a more complex psychological organization. Identifying these profiles can guide clinicians to tailor interventions, especially those aimed at strengthening self-esteem and reducing body-related shame, according to individuals’ specific vulnerabilities.

The present findings are based on female patients only. Eating disorders also occur in males, and male presentations can be under detected and may differ in symptom expression and help-seeking pathways; therefore, findings may not generalize beyond females. Future research should test whether similar profiles emerge in mixed-gender and male clinical samples (Gorrell and Murray, 2019). Eating disorders are multi-factorial conditions in which biological vulnerability, psychological processes, and interpersonal and sociocultural factors interact in symptom onset and maintenance. Within this framework, self-esteem, shame, loneliness, and body-related attitudes may shape how distress is experienced and expressed (Calogero, 2009; Stice, 2002; Bulik, 2005). Future research should replicate these findings in larger and more diverse samples and examine how these psychological profiles evolve.

The present study also has several limitations that should be acknowledged. First, there are limitations regarding participants and the generalizability of results. Our findings are based exclusively on a small sample of patients, all female, attending a single outpatient clinic for the treatment of eating disorders, which represent only a limited part of the general population affected by such disorders. Furthermore, the age range of the participants was broad and may have contributed to greater variability. This broad age range may reflect different developmental stages and illness pathways; therefore, results should be replicated in larger samples and, when possible, in age-stratified analyses. Second, some limitations concern the procedure and experimental design of the study. Our conclusions are limited by the cross-sectional nature of the study, which does not allow us to establish causality and directionality of effects. Third, limitations concerning the variables and scales used should be noted. Psychological variables were assessed using self-report instruments, which can introduce several sources of bias. Responses may be influenced by social desirability. Furthermore, these measures also rely on participants’ self-awareness, which may be limited when assessing complex emotional and bodily experiences. In addition, the Body Protection scale showed low reliability, and therefore findings related to this dimension should be interpreted with caution until replicated. Given the overall small sample size, and the small size of one cluster (*N* = 8), the cluster solution may be less stable, and results may be sensitive to sampling variation. Because the number of clustering variables was relatively high compared with our sample, there is also a risk of overfitting and limited reproducibility. Small clusters also reduce statistical power for between-group comparisons and regression estimates; therefore, null findings should be interpreted cautiously and replication in larger samples is needed.

## Data Availability

The raw data supporting the conclusions of this article will be made available by the authors, without undue reservation.
